# A Survey of Autonomous Vehicles: Enabling Communication Technologies and Challenges

**DOI:** 10.3390/s21030706

**Published:** 2021-01-21

**Authors:** M. Nadeem Ahangar, Qasim Z. Ahmed, Fahd A. Khan, Maryam Hafeez

**Affiliations:** 1 School of Computing and Engineering, University of Huddersfield, Huddersfield HD1 3DH, UK; m.ahangar@hud.ac.uk (M.N.A.); M.Hafeez@hud.ac.uk (M.H.); 2 School of Electrical Engineering and Computer Science, National University of Sciences and Technology, Islamabad 44000, Pakistan; fahd.ahmed@seecs.edu.pk

**Keywords:** autonomous vehicles, Bluetooth, dedicated short-range communications, intelligent transport system, V2I, V2V, V2X, VANET, vehicular communications, ultra-wide band, ZigBee

## Abstract

The Department of Transport in the United Kingdom recorded 25,080 motor vehicle fatalities in 2019. This situation stresses the need for an intelligent transport system (ITS) that improves road safety and security by avoiding human errors with the use of autonomous vehicles (AVs). Therefore, this survey discusses the current development of two main components of an ITS: (1) gathering of AVs surrounding data using sensors; and (2) enabling vehicular communication technologies. First, the paper discusses various sensors and their role in AVs. Then, various communication technologies for AVs to facilitate vehicle to everything (V2X) communication are discussed. Based on the transmission range, these technologies are grouped into three main categories: long-range, medium-range and short-range. The short-range group presents the development of Bluetooth, ZigBee and ultra-wide band communication for AVs. The medium-range examines the properties of dedicated short-range communications (DSRC). Finally, the long-range group presents the cellular-vehicle to everything (C-V2X) and 5G-new radio (5G-NR). An important characteristic which differentiates each category and its suitable application is latency. This research presents a comprehensive study of AV technologies and identifies the main advantages, disadvantages, and challenges.

## 1. Introduction

**Figure 1:**
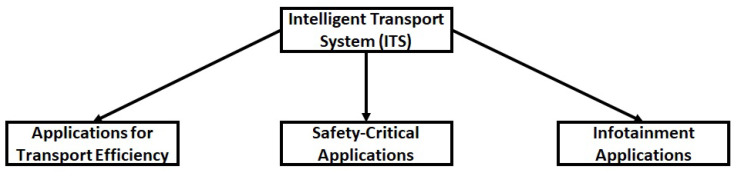
Different applications for intelligent transport system (ITS).

Technology facilitates humans, improves productivity and leads to a better quality of life. Technological developments and automation in vehicular networks will lead to better road safety and lower congestion in present urban areas where the traditional transport system is becoming increasingly disorganised and inefficient [1]. Therefore, the development of the intelligent transport systems (ITS) concept has been proposed, with the aim and focus on improving traffic safety and providing different services to its users [2,3]. There has been considerable research in ITS resulting in significant contributions (see [4] and references therein). Figure 1 shows the three main applications of the ITS system: (1) Applications for transport efficiency; (2) Safety-critical applications; and (3) Infotainment applications. The main task of the transport efficiency application includes the calculation of the optimal speed and the route to navigate the vehicle to the destination by taking into account the traffic information [5]. Safety-critical applications deal with the response to emerging vehicles and intersection collision avoidance [5]. Infotainment applications deal with cooperative local services such as the location of petrol stations, hotels, etc. [5].

**Figure 2:**
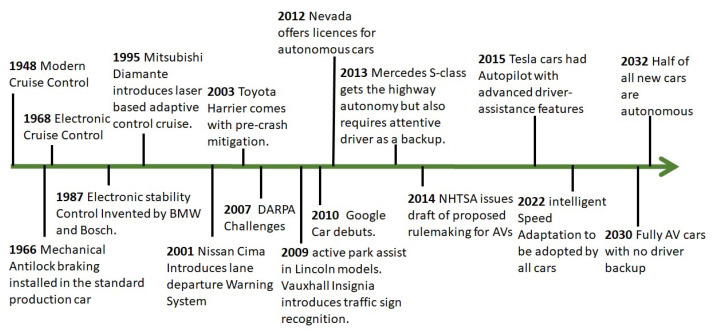
Technical evolution in autonomous cars.

One of the most significant contributions towards the ITS has been the development of autonomous vehicles (AVs). These vehicles perceive the environment through sensing using various sensors, and then this information is utilised to drive without the need for any human intervention [6]. In Figure 2, the technical evolution of AVs is illustrated in detail. The modern cruise control system was developed in 1948 and since then self-driving cars have been gradually developing different features [7,8]. Significant developments were made in the early 2000s when the lane departure warning system (LDWS), adaptive cruise control (ACC), self-parking assistance (SPA), auto-pilot and traffic sign recognition (TSR) were developed for AVs [9,10,11]. It is planned that by 2022 we will have intelligent speed adaptation systems and by 2030 fully automated AVs will be available with no driver backup required. This advancement will further lead to the next emerging and evolutionary stage of the Internet of vehicles (IoV). In IoV networks, besides human as users of the Internet, physical sensors, machine-type devices and vehicles will be the users of the IoV ecosystem.

The Society of Automotive Engineers (SAE) classified the vehicles into six different levels of automation. The same classification is also adopted by other organisations such as the International Organisation of Motor Vehicle Manufacturer (OICA) and the German Federal Highway Research Institute (BASt). The US National Highway Traffic Safety Administration (NHTSA) initially had a different classification, as compared in Table 1 [12]. Since then, NHTSA has also adopted SAE’s six levels of automation in the Federal Automated Vehicle Policy. Levels 0–2 can be broadly categorised as driver assisted, while Levels 3 and 4 can be termed as semi-automatic and Level 5 is fully autonomous. The detailed features of each level are described below:

- Level 0 is not automated, and all the tasks are performed by the driver.
- Level 1 provides driver assistance with some functions such as ACC, TSR, etc., but the driver controls the accelerator and the brakes while monitoring the surroundings.
- Level 2 provides partial driving assistance, and the vehicle can perform the steering and acceleration functions. However, the driver is responsible for many safety-critical actions.
- Level 3 provides conditional driving automation where the vehicle performs the entire monitoring of the surroundings. The driver is no longer responsible for safety-critical issues.
- Level 4 provides high driving automation, and the driver holds control only if the automated situation turns unsafe. Steering, braking, acceleration and surrounding check are performed by the vehicle.
- Level 5 represents complete automation where there is no need for human intervention and the driver is a passenger.

**Figure 3:**
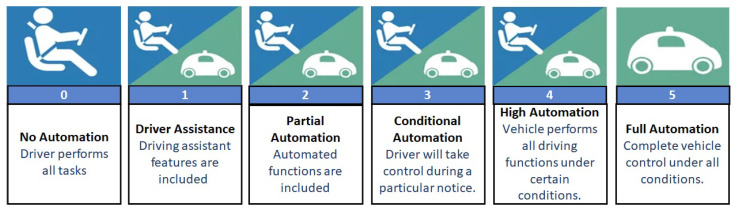
Different levels of automation.

Figure 3 summarises the above discussed features and functions achieved at each level according to the SAE J3016 standard [12].

Autonomous vehicles have several advantages including improved safety and lower road congestion resulting in lower fuel/energy consumption [4,6]. Besides these advantages, there are some issues which need to be resolved for AVs such as who bears the legal responsibilities of the AVs, what will the course of action be if the AV controller is hacked, etc. A summary of the main pros and cons of AVs are listed in Table 2. Overall, it can be claimed that, if the drawbacks are resolved or minimised, then autonomous cars will be a considerable technological development in coming years as they will facilitate people’s lives and increase road safety [10,13].

This paper aims to review the advancement of autonomous cars focusing on the various sensors deployed in them along with the different communication technologies employed. Therefore, the objectives of this survey are as follows:

- To identify and describe the different type of sensors used in AVs.
- To classify communication technologies based on their transmission range and identify their main advantages and disadvantages. This implies:
  1. Discussing short-range technologies such as Bluetooth, ZigBee and ultra-wide band (UWB)
  2. Assessing medium-range technologies such as dedicated short-range communication (DSRC) and Wireless Fidelity (Wi-Fi)
  3. Analysing long-range technologies such as Cellular Vehicle To Everything (C-V2X) and Fifth Generation-New Radio (5G-NR) technology

The survey is organised as follows. Section 2 presents the literature review. Section 3 discusses the system architecture for the AVs with emphasis on the various types of sensors. Section 4 analyses the Vehicular Ad-hoc Networks (VANETs) and the Vehicle-to-Everything (V2X) technologies. Section 5 discusses the classification of the different wireless technologies based on the transmission range and their applications and challenges in AVs and ITS. Finally, in Section 6, the conclusions and future research directions, along with the decision and planning strategies are presented.

## 2. Literature Review

The focus of the paper is to cover the number of sensors employed for AVs and the virtual server for data transmission. Most applications for ITS require a multitude of sensors, which assist automation and communication. These are mounted within a vehicle or on the network infrastructure. Depending on the transmission range of the wireless technology, the sensors are divided into three main categories, namely short-range, medium-range and long-range. Each category has a separate communication latency and, thus, is deployed in different scenarios. A summary of the wireless technologies, their categorisation and use cases in existing literature are referenced and discussed in Table 3.

In [14], the architecture design and implementation of an AV is discussed, and, in [15], various strategies of multi-sensor fusion are discussed. The recent developments and advancements in the perception and sensor technologies for AVs are presented in [16]. In [17], multiple-target and multiple-source are combined to form a framework for on-board sensors. In [1], a five-layer architecture for AVs is discussed. However, none of these survey papers discuss the key wireless technologies for data communication. In [18], resource allocation schemes for DSRC and C-V2X are discussed. However, both these technologies are suitable only for medium- and long-range communication and lack low-latency application.

This paper contributes to the current state-of-the-art by providing a holistic review of the key enabling wireless technologies for ITS. It not only identifies the main aspects of network architecture behind AVs, but it also analyses the evolution of the technological trends, the challenges and the constraints that AVs must address before being fully implemented. The paper links abstract technological concepts with the expected real implications that AVs can have in human mobility, transportation systems and infrastructure. Most of the recent papers address these issues separately and they focus on technological innovations, adoption or regulatory issues. Therefore, this paper tries to gather the conceptual frameworks with the practical aspects by illustrating the main architecture concepts and compiling the outcomes of some of the latest research about VANET technologies, including their applications, advantages and limitations.

For short-range communications where transmission range is less than 25 m, four technologies, namely, Bluetooth [19], Bluetooth low energy (BLE) [20], ZigBee [21] and UWB [22], have been proposed. Several papers have been published which compare these wireless technologies, evaluating their main features, such as power consumption, data rates and transmission time (see, e.g., [23,24] and references therein).

For medium-range communication, where transmission range is between 25 and 100 m, WiFi and DSRC are useful wireless technologies [25,26]. However, IEEE 802.11p/DSRC is the prevailing technology for vehicular communications as it supports high-mobility. In addition, another benefit of the IEEE 802.11p/DSRC is the outside-the-context of a basic service set (OCB) mode [27,28]. The OCB mode allows communication without authentication/association and as a result offers lower latency which is necessary for safety-critical applications. However, this may result in severe security issues [27].

Finally, for the long-range communication, from a 100 m to 5 km, Third Generation Partnership Project’s (3GPP’s) long-term evolution (LTE)-Advanced (LTE-A) pro with its Cellular-V2X capabilities and 5G-NR have emerged as the key technologies [29,30,31,32,33]. These technologies provide ubiquitous coverage and high data rates and can support low-to-medium latency requirement. However, they are not suitable for safety-critical applications because the latency varies between 50 and 80 ms depending on the cellular traffic load [34].

**Table 3:** Related work.

Sichitui et al., 2008 [23]	The network protocols from the physical to the transport layer are analysed, and the security aspects for V2V communication are mentioned in detail.
Zeng et al., 2009 [25]	DSRC is developed for V2V and V2I communication. Characteristics, mechanism and the application of DSRC are explained. Security threats and congestion issues are discussed.
Sevlian et al., 2010 [35]	GPS enabled channel sounding system that can predict the multipath and doppler shift for an adaptive OFDM vehicular communication system is developed. 5.9 GHz results in serious multipath and doppler shift issues as compared to the results obtained at 700 MHz band.
Dorle et al., 2010 [36]	ZigBee technology is combined with infrared sensors to detect and validate the vehicles on the intersections. They proposed model utilised ZigBee technology to help the users to recognise and identify the vehicles for security clearance.
Kenney 2011 [26]	Development and deployment of DSRC standards (IEEE 1609.x) is carried out in the United States. Amendments to IEEE 802.11p for wireless access in vehicular environment (WAVE) is proposed for network services, security and multi-channel operations.
Eenennaam et al., 2012 [28]	Channel switching is proposed by IEEE 1604.4 will allow the DSRC technology to support multi-channel operations. Analysis on beaconing with the OMNeT++ simulator is presented.
Habib et al., 2013 [29]	First time classification based on range such as “short-range”, “medium-range” and “long-range” is done considering the available communication technologies.
Jo et al., 2014 [37]	Distributive system for an AV is proposed which reduces computational complexities and modularity. AUTOSAR software application system is proposed.
Zheng et al., 2015 [38]	DSRC and LTE technologies are combined to produce the heterogenous vehicular network (HetVNet) model. HetVNet models combines the advantages of both DSRC and LTE technologies. Characteristics of MAC and network layers are also proposed for this model.
Cerio et al., 2015 [39]	Proposed Bluetooth model is validated by over several road experiments in which the vehicle is supposed to move at high speeds and establish connection with its road-side units.
Gao et al., 2016 [40]	DSRC experimental demonstration for truck platooning is done. Different dynamic and static tests revealed that on hilly and curved roads packet delivery ratio is affected. Lower delivery ratio on straight paths is achieved due to the reflection of the truck with nearby vehicles.
Abboud et al., 2016 [30]	C-V2X and DSRC are combined together to produce a hybrid architecture. It is concluded that the combination of both technologies can produce useful results in V2X communications.
Lei et al., 2016 [41]	ZigBee technology was tested and implemented for Forward Collision Warning System (FCWS) in low speed vehicles.
Zanchin et al., 2017 [12]	Categorising the levels of automation by explaining each level according to the SAE standard.
Zong et al., 2018 [14]	Perception and planning stages can be improved by implementing two inner loops for simultaneously localising and mapping. To enlarge the detection and range of the sensors, they also developed an algorithm to detect and calculate the range of the system.
Fitah et al., 2018 [42]	Demonstrations on various simulators such as SUMO and NS-2.35 to prove the capacity of DSRC technology. After conducting different experiments they concluded that the performance of DSRC is much better than the Wi-Fi for medium-range communications.
Valgas et al., 2018 [31]	3GPP calibrated simulator to evaluate the performance of 5G-NR in V2X communication. An increase in sub-carrier spacing is suggested to boost the system protection against interference.
Rosique et al., 2019 [43]	The physical fundamentals, principal functioning and electromagnetic spectrum of sensors that are used to gather the external information of the vehicle are presented. The sensors are analysed on spider charts in order to compare and highlight their weaknesses.
Sheikh et al., 2019 [44]	VANETs security, architecture and challenges are discussed for traffic management system for vehicular network from malicious nodes and fake messages.
Naik et al., 2019 [32]	IEEE 802.11bd and 5G-NR are developed to overcome the disadvantages of DSRC and C-V2X technology and satisfy the diverse needs for V2X applications.
Chen et al., 2020 [33]	Basic road safety, architecture, key technologies and the standards of C-V2X are introduced. Technical evolution path from LTE-V2X to NR-V2X is covered.
Noor et al., 2020 [18]	Fair allocation of resources is of prime importance in wireless networks [45]. Resource allocation in vehicular networks has been surveyed in [18] and references therein.

## 3. System Architecture for Autonomous Vehicles

**Figure 4:**
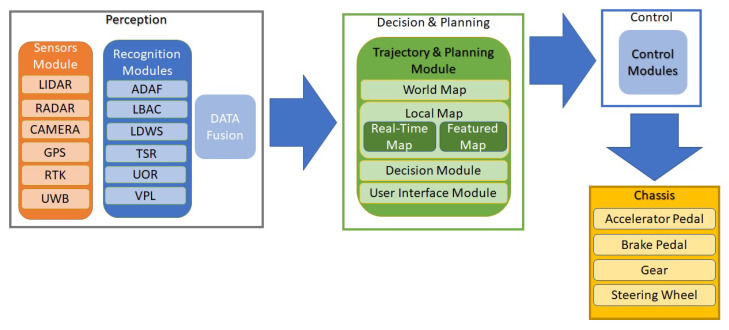
System Architecture for AVs.

An ordinary vehicle can be converted into an autonomous one by adding some additional components including sensors that allow the vehicle to make its own decisions by sensing the environment and controlling the mobility of the vehicle [12,14,37,46]. Figure 4 illustrates the overall communication process/protocol in AVs and also lists the sensors, actuators, hardware and the software control required. The protocol architecture, explained below, is composed of four main stages and enables a Level 5 fully autonomous vehicle where all the users are passengers.

1. Perception: This stage involves sensing of the AVs surrounding through various sensors and also detecting its own position with respect to the surroundings. In this stage, some of the sensors used by the AV are RADAR, LIDAR, camera, real-time kinetic (RTK), etc. The information from these sensors is then passed to the recognition modules which process this information. Generally, the AV consists of adaptive detection and recognition framework (ADAF), a control system, LDWS, TSR, unknown obstacles recognition (UOR), vehicle positioning and localisation (VPL) module, etc. This processed information is fused and passed to the decision and planning stage.
2. Decision and Planning: Utilising the data gathered in the perception process, this stage decides, plans and controls the motion and behaviour of the AV. This stage is analogous to the brain and makes decision such as path planning, action prediction, obstacle avoidance, etc. The decision is made based on the current as well as past information available including real-time map information, traffic details and patterns, information by the user, etc. There may be a data log module that records errors and information for future reference.
3. Control: The control module receives information from the decision and planning module and performs functions/actions related to physical control of the AV such as steering, braking, accelerating etc.
4. Chassis: The final stage includes the interface with the mechanical components mounted on the chassis such as the accelerator pedal motor, brake pedal motor, steering wheel motor and gear motor. All these components are signalled to and controlled by the control module.

After discussing the overall communication and sensor architecture of an AV, we discuss the design, functionality and utilisation of some main sensors.

### 3.1. Ultrasonic Sensors

These sensors use ultrasonic waves and operate in the range of 20–40 kHz [14]. These waves are generated by a magneto-resistive membrane used to measure the distance to the object. The distance is measured by calculating the time-of-flight (ToF) of the emitted wave to the echoed signal. Ultrasonic sensors have very limited range which is generally less than 3 m [47]. The sensor output is updated after every 20 ms [47], making it not compliant with the strict QoS constraints of an ITS. These sensors are directional and provide a very narrow beam detection range [14]. Therefore, multiple sensors are needed to to get a full-field view. However, multiple sensors will influence each other and can cause extreme ranging errors [48]. The general solution is to provide a unique signature or identification code which will be required to discard the echoes of other ultrasonic sensors operating in near-by range [49]. In AVs, these sensors are utilised to measure short distances at low speeds. For example, they are used for SPA and LDWS [50]. Moreover, these sensors work satisfactorily with any material (independent of color), in bad weather conditions and even in dusty environments.

### 3.2. RADAR: Radio Detection and Ranging

RADARs, in AVs, are used to scan the surroundings to detect the presence and location of cars and objects. RADARs operate in the millimetre-wave (mm-Wave) spectrum and are typically used in military and civil applications such as airports or meteorological systems [51]. In modern vehicles, different frequency bands such as 24, 60, 77 and 79 GHz are employed and they can measure a range from 5 to 200 m [52]. The distance between the AV and the object is calculated by measuring the ToF between the emitted signal and the received echo. In AVs, the RADARs use an array of micro-antennas that generate a set of lobes to improve the range resolution as well as the detection of multiple targets [53]. As mm-Wave RADAR has higher penetrability and a wider bandwidth, and it can accurately measure the short-range targets in any direction utilising the variation in Doppler shift [51,52,53,54]. Due to longer wavelength, mm-Wave radars have an anti-blocking and anti-pollution capability that allows them to cope in rain, snow, fog and low-light. Furthermore, mm-Wave radars have the ability to measure the relative velocity using the Doppler shift [15]. This ability of mm-Wave radars make them suitable for extensive AV application such as obstacle detection [55], pedestrian recognition [56] and vehicle recognition [57]. Some applications of RADARs in AVs are forward cross traffic alert (FCTA), lane change assistance (LCA), blind spot detection (BSD), rear cross traffic alert (RCTA), etc. The mm-Wave also has some disadvantages such as reduced field-of-view (FoV), less precision and results in getting more false alarm as a result of emitted signals which gets bounced from the surroundings [54].

### 3.3. LiDAR: Light Detection and Ranging

LiDAR utilises the 905 and 1550 nm spectra [58]. The 905 nm spectrum may cause retinal damage to the human eye, and, therefore, the modern LiDAR is operated in the 1550 nm spectrum to minimise the retinal damage [43]. The maximum working distance of LiDAR is up to 200 m [15]. LiDAR can be categorised into 2D, 3D and solid-state LiDAR [59]. A 2D LiDAR uses the single laser beam diffused over the mirror that rotates at high speed. A 3D LiDAR can obtain the 3D image of the surrounding by locating multiple lasers on the pod [60]. At present, the 3D LiDAR can produce reliable results with an accuracy of few centimetres by integrating 4–128 lasers with a horizontal movement of 360 degrees and the vertical movement of 20–45 degrees [61]. The solid-state LiDAR uses the micro-electromechanical system (MEMS) circuit with micro-mirrors to synchronise the laser beam to scan the horizontal FoV several times. The laser light is diffused with the help of a micro-mirror to create the vertical projection of the object. The received signal is captured by a photo-detector and the process repeats until the complete image of the object is created. LiDAR is used for positioning, obstacle detection and environmental reconstruction [15]. 3D LiDAR sensors are playing an increasingly significant role in the AV system [62]. As a result, the LiDARs can be used for ACC, 2D or 3D maps and object identification and avoidance. A roadside LiDAR system has shown to reduce the vehicle-to-pedestrian (V2P) crashes both at intersections and non-intersection areas [63]. In [63], a 16-line real-time computationally efficient LiDAR system is employed. Deep auto-encoder artificial neural network (DA-ANN) is proposed, which achieves an accuracy of 95% within a range of 30 m. In [64], a 64-line 3D LiDAR utilising a support vector machine (SVM)-based algorithm is shown to improve the detection of the pedestrian. Although LiDAR is superior to a mm-Wave radar in measurement accuracy and 3D perception, its performance suffers under severe weather conditions such as fog, snow and rain [65]. In addition, its operating range detection capability depends on the reflectiveness of the object [66].

### 3.4. Cameras

The camera in AVs can be classified as either visible-light based or infrared-based depending upon the wavelength of the device. The camera uses image sensors built with two technologies that are charge-coupled device (CCD) and a complementary metal-oxide-semiconductor (CMOS) [43]. The maximum range of the camera is around 250 m depending on the quality of the lens [15]. The visible cameras use the same wavelength as the human eye i.e., 400–780 nm, and is divided into three bands: Red, Green and Blue (RGB). To obtain the stereoscopic vision, two VIS cameras are combined with known focal length to generate the new channel with the depth (D) information. Such a feature allows the camera (RGBD) to obtain a 3D image of the scene around the vehicle [67].

The infrared (IR) camera uses passive sensors with a wavelength between 780 nm and 1 mm. The IR sensors in AVs provide vision control in peak illumination. This camera assists AVs in BSD, side view control, accident recording and object identification [68]. Nevertheless, the performance of the camera changes in bad weather conditions such as snow, fog and moment-of-light variation [15].

The main advantages of a camera are that it can gather and record the texture, color distribution and contour of the surroundings accurately [15]. However, the angle of observation is limited due to narrow view of the camera lens [69]. Therefore, multiple cameras have been adopted in AVs to monitor the surrounding environment [70,71]. A three-stage RGBD architecture using deep learning and convolutional neural networks was proposed by Ferraz et al. for vehicle and pedestrian detection [72]. However, this requires the AV to process huge amount of data [15]. Currently, AVs do not possess such computational resources; therefore, computational offloading may be an appropriate solution [73].

Table 4 summarises the challenges of the discussed sensor technologies. It can be observed in Table 4 that the detection capability and reliability of the various sensors is limited in different environments. This limitation can be overcome and the accuracy of target detection along with the reliability can be improved through multi-sensor fusion. Radar–camera (RC) [56,74], Camera–LiDAR (CL) [60,75], Radar–LiDAR (RL) [76] and Radar–Camera–LiDAR (RCL) [77,78] have been proposed where different sensors are combined together to improve the perception of the environment. Furthermore, in [14], three different sensor plans are developed based on range, cost and balance function. In this study, several different sensors are combined. In Plan *A*, four cameras, a mm-Wave RADAR, 32- and 4-layer LiDAR and a GPS+IMU are employed. In Plan *B*, four cameras, three mm-Wave RADAR, a four-layer LiDAR and a GPS+IMU are utilised. Finally, in Plan *C*, two regular cameras, three mm-Wave RADARs, a surrounding camera and a twelve-unit ultrasonic sensor are utilised.

### 3.5. GNSS and GPS, IMU: Global Navigation Satellite System and Global Positioning System, Inertial Measurement Unit

This technology can determine the exact position of the AV and helps it navigate [79]. GNSS utilises a set of satellites orbiting around the earth’s surface to localise [80]. The system contains the information of AV’s position, speed and the exact time [80]. It operates by calculating the ToF between the satellite emitted signal and the receiver [81]. The AV position is usually extracted from the Global Positioning System (GPS) coordinates. The extracted coordinates by GPS are not always accurate and they usually introduce an error in the position with a mean value of 3 m and a standard deviation of 1 m [82]. The performance is further degraded in urban environments and an error in position can increase up to 20 m [83] and in some extreme cases the GPS position error is around 100 m [84]. In addition to this, the RTK system can also be used in AVs to precisely calculate the position of the vehicle [85]. Furthermore, dead reckoning (DR) and the inertial position can also be used in AVs to determine the position and the direction of the vehicle [86]. A technique known as odometry can be used to measure the position of the vehicle by fixing the rotary sensors to the wheels of the vehicle [79]. To make the AV capable of detecting slippage or lateral movements, the inertial measurement unit (IMU) is used and it detects this using accelerometers, gyroscopes and the magnetometer sensor’s data. The IMU combined with all units can rectify the errors and increases the sampling speed of the measuring system. Although the IMU cannot provide the position error unless it is not accompanied by the GNSS system, AVs can get information from different sources such as RADAR, LiDAR, IMU, GNSS, UWB and camera to minimise the possibilities of error and perform reliable position measurement [43]. GPS can be combined with IMU techniques such as DR and the inertial position to confirm and improve the position estimate of the AV [87].

### 3.6. Sensor Fusion

Real-time and accurate knowledge of vehicle position, state and other vehicle parameters such as weight, stability, velocity, etc. are important for vehicle handling and safety and, thus, need to be acquired by the AVs using various sensors [88]. The process of sensor fusion is used to obtain coherent information by combining the data obtained from different sensors [15]. The process allows the synthesis action of raw data obtained from complimentary sources [89,90]. Therefore, sensor fusion allows the AV to precisely understand its surrounding by combining all the beneficial information obtained from different sensors [91]. The fusion process in AVs is carried out by using different types of algorithms such as Kalman filters and Bayesian filters. The Kalman filter is considered very important for a vehicle to drive independently because it is utilised in different applications such as RADAR tracking, satellite navigation system and visual odometry [92].

#### 3.6.1. Kalman Filters

The Kalman filter (KF) is used to calculate the probabilistic state of the dynamic system in the past (smoothing), present (filtering) or future (prediction) [93]. As the sensors used in self-driving cars produce noisy and incomplete data, the role of the Kalman filter is to eradicate the unwanted noise and obtain accurate estimates [94]. The state of the system (x) is composed of the position and velocity of the AV [95]. In addition, as the data are obtained from different sensors such as Radar, Lidar, ultrasonic, etc., the measured/observed position and velocity of the AV varies depending upon the accuracy of these sensors. This uncertainty in the data (of position and velocity) can be reduced by combining the measured data with the prediction of the states utilising the Kalman filter. The Kalman filter models the uncertainty using a Gaussian model [16]. The variance of the Gaussian model, $\sigma^{2}$, measures the amount of uncertainty in the state of the system and thus, a larger variance implies greater uncertainty [96]. To accurately estimate the state of the system, with low uncertainty, the Kalman filter compares the current observation with the previous prediction. The process is recursive unless the measurement data are unavailable.

##### Mathematical Model

The Kalman filter is composed of two stages, prediction and update, to finalise the actual state of the system [93,97]. Considering a dynamic system moving linearly, its mathematical model can be briefly explained as follows.

##### Calculation of the Prediction Stage

In the prediction stage, the state, $x_{1}$, of the system with an uncertainty of $P_{1}$ at time *t*, is estimated from its previous state x and P at time (t−1). The formula to calculate the predicted state is as follows [93]

(1) $$\begin{array}{ccc} x_{1} & = & Fx+n \end{array}$$

(2) $$\begin{array}{ccc} P_{1} & = & FPF^{H}+R \end{array}$$

where F is the transition matrix from (t−1) to *t*, n is the added noise and R is the noise co-variance matrix.

##### Calculation of the Update Stage

In the update stage, the predictions are modified by comparing them with the data obtained from sensors. If z represents the measured data from sensors, then the estimated error between the measurement and the prediction is given as [98]

(3) $$y=z-Hx_{1},$$

where H represents the transition matrix between the measured sensor data [93,99]. Similarly, the estimated system error (E) can be given as $E=HP_{1}H^{T}+R$. The Kalman filter gain (K) is given by $K=P_{1}H^{T}E^{-1}$. The essential purpose of Kalman gain is to correct predictions [99,100]. The actual state of the system and position is predicted as [93]

(4) $$\begin{array}{c} x=x_{1}+Ky \end{array}$$

(5) $$\begin{array}{c} P=\left(I-KH\right)P_{1} \end{array}$$

Even though the measurement will provide data that are originated from sensors, the information in the predicted and measurement states is always uncertain and therefore the optimal state estimate will be the one in which the position has a lower uncertainty [101].

From the above equations, it can be noted that, to learn the vehicle position and state, the knowledge of the transition matrix and the noise co-variance matrix is required. Traditional Kalman filtering yields the optimal solution if the noise statistics and noise process is completely defined [102]. These matrices are configured with priori fixed values that do not adapt with the changing surroundings [103]. Ideally, these matrices should change, adapt and update dynamically according to their surroundings and environment. Therefore, various approaches have been adopted in the literature to adapt the matrices and improve the estimate of the state and the position of the vehicle [104,105].

In this regard, there has been significant research interest in Extended Kalman filters (EKF), Unscented Kalman filters (UKF) and Cubature Kalman filters (CKF) [105,106,107]. In [108], the EKF was used to estimate the lateral and longitudinal tire forces to improve the handling and safety of the vehicle. In [109], a dual extended Kalman filter (DEKF) was proposed which employs two parallel EKFs to estimate the position and state of the AV. In [110], a DEKF was applied to estimate the road friction parameters. However, EKFs are strongly influenced by the approximated noise and diverge when non-linearity of the model dominates [111]. An adaptive EKF (AEKF) was proposed by Akhlaghi et al. [112], which adopts a traditional co-variance matching technique. Both matrices are updated continuously by making the filter residual co-variances consistent with the theoretical co-variances.

A more accurate generalisation is to employ the Unscented Kalman Filter (UKF) [113]. UKF for vehicle dynamics is proposed in [114,115]. A dual UKF (DUKF) has been applied to simultaneously estimate the sideslip angle and vehicle mass [116]. However, in a higher dimension filtering problem, the cubature Kalman filter (CKF) has shown better stability and accuracy [107,117]. The improved performance is because of its ability to solve the multi-dimensional integral. Furthermore, in [118], the dual CKF (DCKF) is proposed, which performs better than the CKF under various friction coefficients when estimating the position and states of the AV. The UKF and CKF fall in the sampling based KFs and the performance of these algorithms depends upon the accuracy of instantiated sigma points [105]. Therefore, Particle filter (PF) and its variants, which are discussed in next section, are ideal for non-linear and non-Gaussian problems.

#### 3.6.2. Particle Filters

The basic assumption of the Kalman filters is that the noise experienced by the system is Gaussian [101]. However, in most cases, the noise experienced by the system will not have a Gaussian characteristic [119]. Particle filters (PF) have been proposed for processing non-Gaussian noise (see, e.g., [120] and the references therein). When applying PF there is always a trade-off between precision and computational resources [111,121]. Therefore, an extended particle filter (EPF) [122] and an unscented particle filter (UPF) [111] have been proposed. Both EPF and UPF use the sigma points, making them computationally inefficient and difficult to implement in real time [122]. Recently, particle-aided unscented Kalman filter (PAUKF) has been proposed [101] which does not update the sigma points resulting in fewer computational resources. For vehicle localisation, the PAUKF algorithm has been shown to perform consistently better compared to existing methods including PF and UKF and yields an RMSE of approximately 1.5 m [101].

## 4. Vehicular Ad-Hoc Networks (VANETs)

VANETs are an emerging sub-class of mobile ad-hoc networks capable of spontaneous creation of a network of mobile devices/vehicles [123]. VANETs can be used for vehicle-to-vehicle (V2V) and Vehicle-to-Infrastructure (V2I) communication [44,124]. The main purpose of such technology is to generate security on the roads; for example, during hazardous conditions such as accidents and traffic jam the vehicles can communicate with each other and the network to share vital information [125,126]. The main components of VANET technology are:

1. On-board unit (OBU): It is a GPS-based tracking device embedded in every vehicle to communicate with each other and with roadside unit (RSU) [124,126]. To retrieve the vital information, the OBU is equipped with many electronic components such as resource command processor (RCP), sensor devices and user interfaces. Its main goal is to communicate between different RSUs and OBUs via a wireless link [44].
2. Roadside Unit (RSU): RSU is a computing unit fixed at specific location on roads, parking areas and intersections [127]. Its main goal is to provide connectivity between autonomous vehicle and the infrastructure and also assists in vehicle localisation [44,127]. It can also be used to connect vehicle with other RSUs using different network topologies [44]. They have also been powered using ambient energy sources such as solar power [128].
3. Trusted Authority (TA): It is an authority which manages the entire process for VANETs, so that only valid RSUs and vehicle OBUs can register and communicate [129]. It provides security by verifying the OBU ID and authenticates the vehicle. It also detects malicious messages or suspicious behaviour [44].

VANETs have some unique properties which are very different from other ad-hoc technologies.

- VANETs have very low discovery latency and as a result the vehicles, even at high speeds, connect to the RSU quickly and rarely face network outage [130,131].
- The OBUs can move with predictable and regular path. It can help to detect the actual trajectory of the vehicle at any point of time [131]. The RSUs in VANETs can localise the vehicle and also log the path of the vehicle and also predict its trajectory to avoid any hazard.
- The vehicle sensors and other nodes do not face any energy restrictions because they can extract energy from the vehicle engine.
- The use of multicast broadcasting in VANETs allows the different vehicles to communicate with each other simultaneously [132].

**Figure 5:**
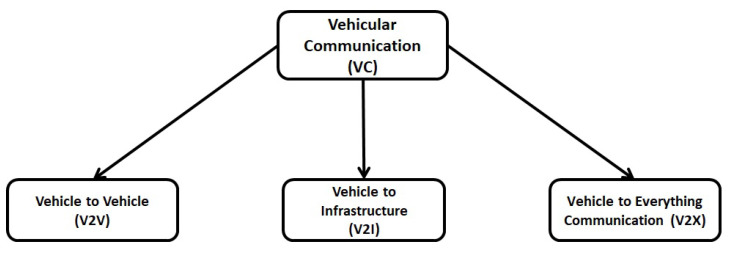
Vehicular communication (VC) system.

Vehicular communication, utilising VANETs, includes V2V communication, V2I communication and V2X communication, as illustrated in Figure 5. The details are given below.

### 4.1. Vehicle-To-Vehicle (V2V) Communication

It is also called inter-vehicle communication (IVC) that allows the vehicles to communicate with each other and share the necessary information about traffic congestion, accidents and speed limits [4]. V2V communication can generate the network by connecting different nodes (Vehicles) using a mesh (partial or full) topology [5]. Depending upon the number of hops used for inter-vehicle communication, they are classified as single-hop (SIVC) or Multi-hop (MIVC) systems [9]. The SIVC can be used for short-range applications such as lane merging, ACC, etc., whereas MIVC can be used for long-range communication such as traffic monitoring. The V2V communication provides several advantages such as BSD, FCWS, automatic emergency braking (AEB) and LDWS [4].

### 4.2. Vehicle-To-Infrastructure (V2I) Communication

It is also known as roadside-to-vehicle communication (RVC) and allows the vehicles to interact with the RSUs. It helps to detect traffic lights, cameras, lane markers and parking meters [1]. The communication of vehicles with the infrastructure is ad-hoc, wireless and bidirectional [10]. The data collected from the infrastructure are used for traffic supervision and management. They are used to set different speed variables allowing the vehicles to maximise fuel efficiency as well as control the traffic flow [4]. Depending on the infrastructure, the RVC system can be divided into the Sparse RVC (SRVC) and the Ubiquitous RVC (URVC) [23]. The SRVC system provides communication services at hotspots only, for example to detect available parking spaces or gas stations, whereas the URVC system provides coverage throughout the road, even at high speeds. Therefore, the URVC system requires a large investment to ensure network coverage [23].

### 4.3. Vehicle-To-Everything (V2X) Communication

The V2X communication allows the vehicle to communicate with other entities such as pedestrians (V2P), roadside (V2R), devices (V2D) and the Grid (V2G) [133]. This communication is used to prevent road accidents with vulnerable pedestrians, cyclists and motorcyclists [20]. The V2X communication allows the Pedestrian Collision Warning (PCW) mechanism to alert the roadside passenger before any serious accident takes place. The PCW can access the Bluetooth or Near Field Communication (NFC) of the smartphone and may use beacon stuffing to deliver critical messages to the pedestrian [4].

## 5. Different VANET Technologies Based on Transmission Range

**Figure 6:**
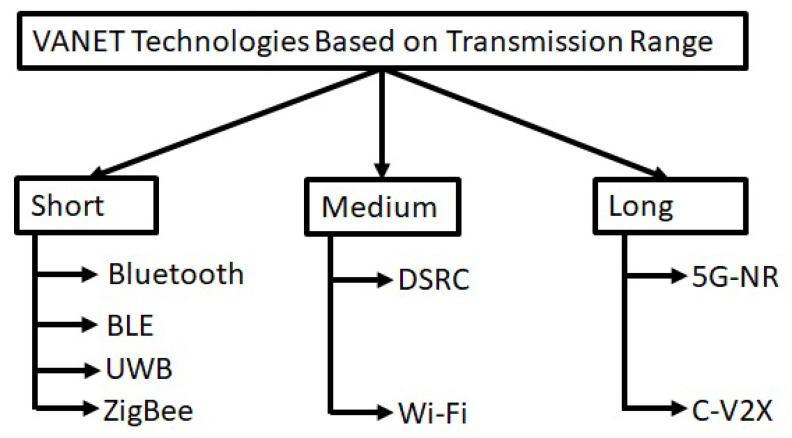
Different VANET technologies considered for AVs.

An AV can communicate with other vehicles and the network infrastructure by using different wireless technologies. For example, the AV may transmit and receive real-time data (Audio/Video) during V2X communication or in case of emergency situations such as fog or accidents, warning information can be exchanged with neighbouring vehicles in a short time [29]. As discussed above and shown in Figure 4, the wireless technology employed depends on the system architecture and particularly on the kind of sensors employed along with the use case. The wireless technologies used in autonomous vehicles can be differentiated in terms of their transmission range, as shown in Figure 6.

The long-range technologies allow the vehicles to transmit data while being miles apart; examples are C-V2X and 5G-NR communication. The medium-range communication, using Wireless Local Area Network (WLAN) or DSRC, can provide the coverage for V2V and V2I communication within a range of tens or hundreds of feet. The short-range communication technologies include Bluetooth, UWB and ZigBee. Below, these wireless technologies are discussed in detail.

### 5.1. Short-Range Communication Technologies

#### 5.1.1. Bluetooth Technology

The Bluetooth technology based on IEEE 802.15.1 protocol can be used as the short-range communication technology in the vehicular network [20]. It operates in the 2.4 GHz band and can produce the data rate up to 1–4 Mbps with variable coverage ranges [134]. The range of Bluetooth varies due to the propagation conditions, the sensitivity and gain of the transmitting and receiving antennas [39]. Wi-Fi and Bluetooth use the same bandwidth; therefore, to avoid interference, Frequency Hopping Spread Spectrum (FHSS) is used in Bluetooth [135]. Different versions of Bluetooth use different standards. For example, Bluetooth 4.0 (BLE) uses low energy for data transmission, but it is not compatible with older versions of Bluetooth known as “Bluetooth classic” (BC) [136].

Due to the low transmission range of BLE, it is unsuitable for inter-vehicular communication [137]. However, the range and throughput supported by BLE make it a suitable candidate to eliminate the complex wiring within an AV and reduce its weight. BLE is suitable for wireless audio stream transmission and can be used to connect the AV’s infotainment system wirelessly to the speakers and microphone. In addition, it may be used to wirelessly connect sensors to the ECU and OBU and reduce the cost and complexity of wiring. In [138], BLE was compared to the CAN bus for intra-vehicular communication and it was shown that the BLE is a suitable low cost energy efficient solution for communication with less critical sensors having relaxed latency and throughput requirement. However, as BLE takes time to discover devices and establish connection, it is not suitable for critical delay sensitive vehicular applications [139]. In [140], an algorithm utilising the BLE technology is proposed for indoor positioning and exterior localisation for vehicular applications. Furthermore, Bluetooth vulnerabilities are highlighted in [141]. Blueborne, which is identified in [142], can have a serious effect on automotive security. However, detailed Bluetooth and BLE vulnerabilities for AVs are still missing in the literature [143].

#### 5.1.2. ZigBee Technology

ZigBee technology was developed to address the need for low cost and low power wireless Internet-of-things (IoT) network [144]. The supported connectivity range is up to 100 m with a transfer data rate of about 250 kbps [145]. It is also recognised as IEEE 802.15.4 for Low Rate Wireless Personal Area Network (LRWPAN) and uses different frequency bands (868 MHz, 902–968 MHz and 2.4 GHz) for operation [146,147]. ZigBee employs binary phase shift keying (BPSK) or orthogonal quadrature phase shift keying (OQPSK) for modulation.

Similar to BLE, ZigBee also is suitable for intra-vehicular communication and to reduce the complex wiring within an AV. Recently, Volvo deployed a ZigBee based wireless sensor network in trucks to monitor the tire pressure [148]. It was concluded that ZigBee is sufficiently robust for non-safety and non-security critical applications requiring relaxed latency with update rates less than 10 Hz. Pawade et al. proposed the use of ZigBee for Advanced Driver Assistance Systems (ADAS) [149]. In [150], the performance of ZigBee was simulated in the QualNet simulator. It was observed that as the number of cars increased to 50, the packet delivery ratio dropped significantly and the average end-to-end delay was more than 200 s. As a consequence, it was concluded that ZigBee cannot be employed for urban or crowded places. Furthermore, ZigBee technology has successfully been implemented and tested for FCWS in vehicles [41]. ZigBee technology has been successfully implemented to locate monitored vehicles on the real time in [151]. However, the distance promised is based on received signal strength indicator (RSSI) which is not very accurate.

#### 5.1.3. Ultra-Wide Band (UWB) Technology

UWB is considered as the short-range wireless technology using short pulses of bandwidth 3.1–10.6 GHz [22]. Using UWB, the devices can operate at very low power and also support multiple users with data rates greater than 480 Mbps [152,153,154]. With UWB, it is possible to communicate with a unique randomising code by transmitting Gigabits of information per second. These signals have low amplitude that makes them more covert and harder to degrade in the noisy environment [152,153,154]. This allows the UWB to be more secure in transmission with low probability of detection and interception. Devices using the UWB technology are generally designed to have a large processing gain [152,153,154].

Several UWB nodes were deployed in the PRoPART project for obtaining an accurate and robust positioning technique. The proposed solution utilised the inertial and odometry data of the vehicle along with the UWB ranging estimates to estimate the vehicle position. The estimates obtained were accurate and 95% of the computed positions were shown to have a positioning distance error of less than 27.1 cm [155]. In [156], machine learning was employed to improve the RMSE of UWB based ranging estimates, and the positioning distance error was reduced to less than 10 cm. The UWB transmitter employs low power signals which are below −41.3 dBm/MHz emission limit. This low emission with time hopping (TH) code, direct sequence (DS) code or a combination of DS/TH codes protect the data from eavesdropping attack.

A comparison of these four technologies is given in Table 5.

#### 5.1.4. Applications

The short-range communicating technologies can assist various vehicular applications. Some of the application are listed below.

- Vehicle Localisation: The GPS used in the vehicles can easily determine the position of the vehicle but in the dense urban environment its localisation becomes more challenging. However, these short-range technologies, especially UWB, can assist localisation in dense environments as they do not require the Line of Sight (LoS) and can easily penetrate in the obstacles [155]. UWB can be regarded as the technology of choice for range-based localisation especially for the vehicles moving in dense clustered environments [157].
- Real Time Driving Assistance: These short-range communication technologies can be utilised for communication between RSUs and the vehicle’s OBU for gathering of real-time driving data which may help in lane assist and avoidance of road accidents [155].
- Vehicle Identification: As already discussed, these technologies consumes less energy; therefore, they can be useful to implement at toll plazas where the vehicle identification and classification plays a crucial role for tax collection [36].
- Forward Collision Warning Under Low Speed Circumstances: These short-range technologies can be used for short-range V2V communication in low speeds, which can be used to prevent collisions between the self-driving vehicles or for lane and parking assist. This technology offers the best solution to avoid forward collisions between self-driving cars at low speeds [41].
- Others: The above-mentioned technology can provide additional advantages such as identifying the car owner, detecting the position of passengers in the vehicle and deactivating the air bags on emergency cases [140,147,155].

### 5.2. Medium-Range Communication Technologies

#### 5.2.1. Dedicated Short-Range Communication (DSRC)

DSRC or wireless access in vehicular environment (WAVE) is a standard specifically proposed for reliable communication between vehicles and the network infrastructure. The technology can be deployed in OBUs and RSUs to help the vehicles to communicate effectively and create a wide area network [42]. The WAVE/DSRC is a modified version of the Wi-Fi technology (IEEE 802.11), and its set of standards such as IEEE 802.11p and IEEE 1609.x provides some useful results while applying for vehicular access. The interference between DSRC and Wi-Fi can be avoided by setting the frequency spectrum in DSRC much higher than the Wi-Fi spectrum. The difference between Wi-Fi and DSRC is given in Table 6.

DSRC is utilized in V2V communication where OBUs of vehicles communicate with each other. It can also be deployed for V2I communication, where vehicles get assistance from the network infrastructure regarding traffic signals, accident alerts, etc. [25,26]. In addition, the Quality of Service (QoS) of DSRC communication can be improved by using the enhanced distribution channel access (EDCA) protocol [38]. DSRC security threats were classified into three levels: critical, major or minor [158]. Denial of service attack on DSRC was studied by Biswas et al. [159] and its potential countermeasures were analysed by Whyte et al. [160]. Finally, it was argued that DSRC cannot be the standalone system for ITS and a Het-Net approach would be required by Dey et al. [161].

#### 5.2.2. Applications

DSRC technology enables various applications in V2V communication and V2I communication. Some applications are discussed below.

- Traffic Safety: DSRC technology can be used to improve traffic safety [162]. It allows vehicles to detect the barriers and to avoid collision accidents on the roads [35]. With the help of DSRC, the vehicle can detect a sharp curve and warn other cars [27]. If an accident happens on the road, the vehicles can transmit an alert message to other vehicles to avoid collisions [28].
- Traffic Management System: DSRC can help establish a traffic management system which includes highway fleet management, safe overtaking, etc. [40]. The vehicles can safely overtake and maintain a desired gap during lane changes [30]. If a particular vehicle wants to change its lane, it will send the request to other vehicles for lane change [14]. Therefore, the vehicle can perfectly manage the traffic using DSRC communication.
- Vehicle Management: DSRC can be used to identify registered vehicles and provide an automatic pass to them. Hence, trespassing with a non-registered vehicle can be blocked [31].

#### 5.2.3. Limitations

The DSRC technology has several benefits such as low latency, secure transmission and fast network acquisition [163]. The QoS of DSRC is much better than Wi-Fi but the technology itself faces some technical difficulties [42]. Some technical challenges in DSRC technology are mentioned below.

- Hidden Terminal Problem: As the MAC layer consists of two parts, the IEEE 802.11p forms the lower layer while as IEEE 1609.4 contains the upper MAC layer. In DSRC technology, when the packets are transmitted, the ACK mechanism does not exist. In addition, The clear-to-send mechanism is not set by default in carrier-sense multiple access with collision avoidance (CSMA/CA) medium access, which results in the hidden terminal problem [164].
- Congestion Issue: As the number of AVs increase, the communication load also increases, which causes congestion [164]. This congestion leads to delay and packet loss that affects the performance of the network. The increased number of AVs also increases the intensity of the channel will result is severe degradation of performance.
- Handover issue: A vehicle that travels with high speed has to change several network topologies over time. Two types of handover occur: (1) horizontal handover; and (2) vertical handover. Horizontal handover is the handover of the moving AV from one RSU to another, whereas vertical handover is handover of an AV between different cellular LTE base stations. A vertical handover takes longer time compared to the horizontal handover [30]. This will result into the above-mentioned hidden terminal problem.
- Doppler Spread: The Doppler spread occurs due to the relative motion of transceivers. The relative velocities shift the signal frequency and the signal at the receiver is at a different frequency compared to the broadcast signal. The transmitted signal in DSRC technology faces this Doppler shift phenomenon due to vehicle motion on road which will affect predicting the exact location and position of the AV [35].

### 5.3. Long-Range Communications Technology

#### 5.3.1. C-V2X Technology

The vehicles communicate with their surroundings through the cellular network [165]. The C-V2X technology was introduced by 3GPP release 14, and it was further developed by 3GPP release 15 in order to fulfil the criteria of 5G communications [30]. C-V2X technology offers multiple benefits listed below [24].

- C-V2X is highly reliable and can perform communication between the vehicles at high speed.
- The C-V2X technology can perfectly operate in dense traffic conditions, therefore it overcomes the congestion issues faced by DSRC.
- The C-V2X technology supports both short-range and long-range communication between the vehicles and with the RSU.
- The C-V2X technology is able to provide a 360-degree view non-line of sight (NLoS) sensing.
- C-V2X can support much higher data rates compared to the short- and medium-range technologies discussed and, thus, it is able to support applications which require higher data rates.

The C-V2X technology has two modes for vehicular communication. Mode-3 allows the vehicle to communicate via the base-station while Mode-4 allows the vehicles to communicate with each other directly [166]. Mode-4 uses the PC5 interface at 5.9 GHz while Mode-3 uses the traditional LTE interface [13,167]. In addition, Mode-3 depends on centralised scheduling while Mode-4 is completely distributed. The purpose of direct communication between the vehicles (through Mode-4) reduces the latency requirements, necessary for most of time-critical applications in ITS. The vehicles may switch between the desired modes dynamically [167]. LTE and LTE-A have severe security vulnerabilities; the details are provided in [168] (and references therein). Security threats faced by C-V2X is provided in [169]. Furthermore, countermeasures for C-V2X are discussed in detail in [169,170] (and references therein).

#### 5.3.2. 5G-NR Technology: Evolution of C-V2X Technology

The aim of the 5G-NR standard, developed by 3GPP, is to provide higher data rates, lower latency and enable communication between multitude of devices [171,172]. These requirements correspond to three different use cases. First, there is the massive machine type communication (mMTC), which requires low bandwidth, low energy consumption and high connection density to connect a large number of sensors [173]. Second, there is the enhanced mobile broadband (eMBB) for applications requiring extremely high data rates [174]. Third, there is the Ultra Reliable Low Latency Communication (uRLLC) required for applications that require extremely low delays along with low error rate [31,165,175]. The 5G-NR offers interoperability with the C-V2X technology and, as a result, vehicles in a given geographical region can access both technologies. The gains provided by densification of remote radio heads in cloud radio networks and radio head configuration schemes can further contribute towards ubiquitous coverage [128,176]. The comparison between the two technologies is given in Table 7.

#### 5.3.3. Applications

From practical implementation and performance perspective, 5G-NR V2X technologies have potential to contribute in the following areas of enhancements:

- Security and Privacy are critical for successful AV operation in practical settings and largely depend upon the security and privacy provisions of the enabling communication technology. To this end, it is important to ensure the security of one-to-one interactions, e.g., V2N, V2I, etc., as well as one-to-many interactions such as I2V, V2V, etc. In 5G, different approaches such as session based authentication and authorisation can be utilised in the former case; however, for the latter, signing each packet uses a trusted hardware security module can be used for enhanced security [177].
- Management and Orchestration (MANO) have a direct influence on the network performance and hence require optimisation. 5G-NR V2X network infrastructure comprises of various components distributed across the network at various hierarchical levels. The orchestration of such infrastructure allows coordination across all the different network components that are involved in the life-cycle of each network resource block. It involves managing services enabling deployment automation, maintenance, management, configuration, upgrade and repair of each component of the system [177]. ETSI open source MANO (OSM), Network Function Virtualisation (NFV) and Software Defined Networking (SDN) are studied in [178] from V2X perspective for MANO.
- Quality of Service (QoS) has stringent constraints in V2X networks including latency, capacity, etc., as explained above. Several procedures, such as network-application negotiation, connectivity establishment, mobility and user plane management, etc. directly impact the service delivery and its quality. URLLC and eMBB are enabling features offered by 5G for V2X aiming at providing desired QoS to the users.
- Edge services: 5G offers a unique provision of providing computational resources and storage at the network edge. This enables the network to host applications closer to the AVs resulting in reduced latency. Multi-Access Edge Computing (MEC) utilises the network core and backhaul efficiently, therefore reducing the latency requirement of the AVs [178]. Since vehicular users are highly mobile, frequent handovers can be supported via resource management at the edge of the access network [177].

#### 5.3.4. Field Testing

Numerous pilots and feasibility studies have been carried out during the previous decade or so to evaluate the practical performance and suitability of AVs. A comprehensive yet non-exhaustive list of different trials and testing facilities of AVs can be found in [179] for the UK and [180] in Europe and elsewhere. The V2X technologies and communication interfaces are currently in active testing. The US Department of Transport (DoT) is running US’s Safety Pilot Model Deployment [181] as well as NYCDOT [182], THEA DOT [183] and WY DOT [184] pilots that explore DSRC based communication. HarborNet [185] is a test-bed that allows 4G backhauling and Wi-Fi connectivity along with DSRC. The testbed allows cloud-based code deployment, remote network control and distributed data collection from moving container trucks, cranes, tow boats, patrol vessels and roadside units. Google’s automotive driving and testing facility [186] has conducted extensive road operation testing over the last few years. Tesla, Tencent and Baidu have used simulators and road-testing methods together to conduct extensive testing in demonstration zones [187]. 5GIC test bed in Surrey, UK and Espoo and Ispra trials sites in Italy are being utilised for 5G-NR V2X testing [188].

Finally, the wireless technologies which have already been implemented in AVs are discussed in Table 8.

## 6. Conclusions and Future Research Directions

Due to the increase in road traffic, the possibility of accidents and collisions is also increasing. The main goal of the development of self-driving/autonomous vehicles is to make driving safer and more reliable, as well as reduce congestion. For this purpose, these vehicles need to precisely communicate with other vehicles and their surroundings. This process starts at the perception stage when the self-driving vehicle gather information from the environment using various sensors. Even though the data obtained from the sensors might be inaccurate, the fusion process allows the AVs to synthesise this raw data and obtain accurate results. After analysing the surrounding and making a decision, the ad-hoc network topology enables the AVs to communicate with each other and with the network infrastructure to ensure road safety and traffic flow.

Different types of vehicular communication (V2V, V2I and V2X) performed by AVs use different technologies. This survey differentiates these technologies on the basis of transmission range. As a result, Bluetooth, BLE, ZigBee and UWB are grouped as short-range technologies, while DSRC and Wi-Fi are considered medium-range technologies. Similarly, C-V2X and 5G-NR are identified as long-range technologies.

This survey has compared all these technologies with the aim of identifying the applications of V2X communication where each technology is best utilised. For short-range communication technology, this survey discusses its key features and proposes its use for some V2X applications which do not have strict latency requirements such as forward collision warning, toll check and vehicle identification. However, they cannot support applications such as remote driving, remote maintenance, etc. due to their lack of data rates and latency. Out of the four short-range technologies, UWB stands out because it can produce the highest data rate and low latency, and, hence, it can be used for above mentioned vehicular applications.

Medium-range technologies such as DSRC and Wi-Fi can provide higher mobility to the AVs. This survey discussed why DSRC technology provides higher flexibility and is better suited for V2X communication compared to Wi-Fi. Furthermore, the DSRC technology facilitates AVs with different applications such as collision avoidance, lane changing assistance, etc. However, DSRC technology has certain limitations such as congestion issue, Doppler spread and hand-over issue. Based on these limitations and the ultra-low latency requirements, the paper concludes that DSRC technology does not completely fulfil the diverse needs for V2X mobility.

Finally, the survey presents long-range technologies and identifies that C-V2X technology and the 5G-NR are enabling technologies for future intelligent transport systems. This survey discusses the two modes for C-V2X operations: Mode 3 and Mode 4. Mode 3 works within cellular coverage and Mode 4 is suitable for direct V2V communication using the PC5 interface. Moreover, different vehicular applications require high dates, low latency and reliable communication and the 5G-NR technology is able to provide massive machine type communication (mMTC), enhanced mobile broadband (eMBB) and ultra-reliable low latency communication (uRLLC) and, hence, it is suitable for V2X communication. In conclusion, this survey identifies 5G technology as one of the important advancements in the development of autonomous cars and can serve as a backbone of future intelligent transport systems.

### 6.1. Future Research Directions

The deployment of the 5G-NR technology in vehicular communication faces certain technical challenges related to the requirements on the backhaul network, millimetre wave communication and security issues [198]. Recently, the massive number of devices accessing the C-V2X network will cause congestion over the network; therefore, non-orthogonal multiple access (NOMA) is also considered for V2X communication [199]. However, NOMA techniques cannot be straightforwardly applied to C-V2X due to reliability and security constraints. Recently, Riaz et al. [200] (see also the references therein) looked to improve the reliability of NOMA which can be applied to C-V2X or V2V communication. Furthermore, these AVs will help form an Internet of Vehicles (IoV) which will generate big amount of data. Recently, artificial intelligence (AI), machine learning (ML) and deep learning (DL) techniques have been used to improve the state and position estimation and reference therein [15,201,202,203]. Different AI, ML and DL frameworks have been proposed to learn and adapt these matrices online. Techniques such as artificial neural networks (ANNs), adaptive neuro-fuzzy inference system (ANFIS), convolutional neural networks (CNN), deep belief networks (DBN), deep inference for covariance estimation (DICE), long short-term memory (LSTM), recurrent neural networks (RNN) and reinforcement learning have been adopted to estimate the noise co-variance and the transition matrix to improve the position accuracy of the AV [102,203,204,205,206,207,208,209,210]. Further detail and discussion regarding these AI, ML and DL techniques for sensor fusion for AV can be found in [203] (and references therein). Hopefully, all these challenges will be rectified in the future paving the way for AVs and then finally IoVs.

### 6.2. Decision and Planning Strategies

As previously mentioned, self-driving vehicles and AVs must achieve Level 5 automation in order to be able to drive without any human intervention [7]. As a result, vehicles must be intelligent enough to communicate within the traditional transport system and the surrounding [8]. To achieve this, the AVs need to undergo a transition period in which they will have to co-exist with the existing non-autonomous vehicles. Therefore, implementation requires careful and gradual planning both at vehicular and infrastructure level [38].

For the vehicular communication network development, a key factor is generating a heterogeneous model in which different AV communication technologies can be stacked together to perform versatile tasks [40]. For this purpose, DSRC technology seems to be the most appropriate technology for V2V communication, while LTE technology can be used for the implementation of V2I communication. Nevertheless, there are many open issues such as handover, big data management, cross layer design and vehicular cloud computing systems, which still require improvement [38].

Various driving strategies that the AVs must adapt in order to connect and coordinate the information, received from the environment and from other vehicles, are presented in [211]. These driving strategies not only facilitate the implementation of AVs, but also define the basic vehicular competencies needed for traffic flow and safety. They are explained in Table 9.

In addition, there are three main challenges for V2X communication in AVs, which need to be taken into account in any future implementation strategy [212]. Firstly, there is the high cost required for developing and deploying the proper road infrastructure for an ITS. Secondly, VANETs expose critical vehicular information and, thus, require better privacy protection mechanisms. Thirdly, it can be very challenging to build robust VANETs which ensure reliable data transmission between AVs with high mobility and varying location.

Finally, with regards to the infrastructure and environmental aspects, the investment in development of smart cities should incorporate the development of AVs and ITS [213]. In addition, to achieve an ITS, the existing infrastructure is lacking features and requires significant up-gradation, further research and development along with financial investment [214].

## Figures and Tables

**Table 1:** Comparison between different automation levels.

Organisation	Level 0	Level 1	Level 2	Level 3	Level 4	Level 5
BASt, SAE, OICA	Driver Only	Driver Assisted	Partial Automation	Conditional Automation	High Automation	Full Automation
NHSTA	No	Function Specific	Combined Function	Limited Self-Driving	Fully Self-Driving	

**Table 2:** Advantages and disadvantages of AVs.

Advantages	Disadvantages
Casualty: AVs can significantly reduce the number of accidents.	Law: The definition of legal responsibilities can hinder the implementation of AVs.
Fewer Expenses: Precise autonomous driving can reduce fuel consumption and increase the conservation of other parts.	Threat: AVs can be more vulnerable to network hacks because of the present computer-controlled functions.
Productivity: The journey can be productive by performing other activities than driving.	Employment: There will be many job losses due to AVs in the transportation sector.
Comfort: Interiors of AVs can be comfortable and spacious.	Price: The price of AVs is initially high, but, after greater adoption, the price is going to decrease.

**Table 4:** Comparison of sensor and their challenges.

Sensor	Challenges
Ultrasonic Sensors	Cannot be used at high speed Maximum range is 2 m [15] Very low resolution as compared to RADAR
RADAR	Generate a large number of false alarms due to surroundings metal objects The range is between 5 m and 200 m Generated images are of low resolution as compared to Cameras and LiDARs
LiDAR	Suffers extremely from the weather Maximum range is 200 m [15] Very expensive due to high price.
Cameras	Computation overheads which increases the time-critical applications [49] The maximum range is 250 m depending upon the lens [15] A big range resolution gap exists between the Cameras and RADARs or LiDARs.

**Table 5:** Comparison of short-range technologies.

Standards	Bluetooth	BLE	ZigBee	UWB
Specifications	IEEE 802.15.1	IEEE 802.15.1	IEEE 802.15.4	IEEE 802.15.3
Frequency	2.402–2.481	2.402–2.481	868/902–968 MHz, 2.4 GHz	3.1–10.6 GHz
Bandwidth	1	2	0.3/0.6 MHz, 2 MHz	500 MHz–7.5 GHz
Rate	1–3 Mbps	1 Mbps	20–250 kbps	480 Mbps
Range	10 m	50 m	75–100 m	75 m
Latency (msec)	100	6	30	0.1
Modulation	GFSK	GFSK	BPSK,O-QPSK	BPSK-QPSK
Data Protection	16-bit CRC	24-bit CRC	16-bit CRC	32-bit CRC

**Table 6:** Comparison of medium-range technologies.

Parameters	Wi-Fi	DSRC
Specifications	IEEE 802.11a	IEEE 802.11p
Width in channel	20	10
Signalling	OFDM	OFDM
Data Rate (Mbps)	upto 54	upto 27
Modulation type	upto 64QAM	upto 64QAM
Symbol duration (micro sec.)	4	8
Guard Time (micro sec.)	0.8	1.6
FFT Size	64	64
FFT period (micro sec.)	3.2	6.4
Preamble duration (micro sec.)	16	32
Sub carrier spacing (MHz)	0.3125	0.15625
Frequency spectrum (GHz)	5	5.9
Latency (msec)	50	100
Range (m)	100	300

**Table 7:** Comparison of long-range technologies.

Parameters	C-V2X	5G-NR V2X
Subcarrier spacing (kHz)	15	up to 240
Carrier aggregation	Up to 32	up to 16
Channel Bandwidth (MHz)	20	400
Latency (msec)	<10	<1
Reliability	95–99%	99.9–99.999%
Channel Coding	Turbo	LDPC, Polar
Network Slicing	No	YES
Waveform	SC-FDMA	OFDM
Control and data multiplexing	FDM	TDM
Modulation	16 or 64QAM	256 QAM
Communication Type	Broadcast	Broadcast, multicast and unicast
Re-transmission	Blind	PSFCH
Security and privacy	Basic	Advanced
Positioning accuracy (m)	>1	0.1
Frequency Spectrum	800/1800 MHz	700 MHz/3.6 and 26 GHz
Range	100 m to >5 Km	50 m to >5 Km

**Table 8:** Wireless technologies in AVs.

Technology	Task	References
BLE	Wirelessly connect sensors having relaxed latency to the ECU/OBU	[138,139]
	Indoor Positioning and Localisation (On-demand content for the passenger)	[140,189,190]
ZigBee	Forward Collision Warning System	[41]
	Prevent Collision Warning System	[191]
	Wirelessly connect non-safety and non-security critical sensors to the ECU/OBU	[148]
	Advanced Driver Assistance Systems	[149]
UWB	RSU are connected with UWB fixed nodes to improve the position accuracy	[155]
	where the coverage of GNSS is poor. Performance is further improved to 10 cm.	[156]
DSRC	Operational concept for vehicle safety is presented.	[192]
	Field test shows that lane and position of surrounding vehicles can be identified with 100% accuracy when the distance between vehicles is less than 50 m.	[193]
C-V2X	V2X framework is discussed in detail.	[165]
	C-V2X is not able to satisfy all the latency requirements, therefore, shortened transmission time interval is proposed.	[133]
5G-NR V2X	Framework of 3GPP-Version 16 for AV is discussed.	[194]
	Field trial of mm-Wave with 5G-NR, a stable data rate of 2.8 Gbps within 500 ms latency is achieved.	[195]
	Field Trial of 5G-NR V2X satisfying the latency requirements for truck platooning.	[196,197]

**Table 9:** Types of driving strategies.

Driving Strategy	Explanation
Defensive	Holds negative assumptions about its neighbouring AVs.
Competitive	Adopts positive assumptions about its neighbouring AVs.
Negotiated	Negotiates with other AVs based on its implicit decisions.
Cooperative	Communicates with other AVs and accepts unified driving dispatch commands.

## Data Availability

Not applicable.

## Abbreviations

The following abbreviations are used in this manuscript:

2D	2 Dimensional
3D	3 Dimensional
3GPP	3rd Generation Partnership Project
5G-NR	Fifth Generation-New Radio
ACC	Adaptive Cruise Control
ADAF	Adaptive Detection And recognition Framework
AEB	Automatic Emergency Braking
AI	Artificial Intelligence
ANFIS	Adaptive Neuro-Fuzzy Inference System
ANN	Artificial Neural Network
AUTOSAR	AUTomotive Open System ARchitecture
AU	Application Unit
AV	Autonomous Vehicle
BASt	German Federal Highway Research Institute
BLE	Bluetooth Low Energy
BPSK	Binary Phase Shift Keying
BSD	Blind Spot Detection
CCD	Charge Coupled Device
CCH	Control CHannel
CNN	Convolutional Neural Networks
CMOS	Complementary Metal Oxide Semiconductor
CRC	Cyclic Redundancy Check
CSMA/CA	Carrier-Sense Multiple Access with Collision Avoidance
C-V2X	Cellular Vehicle To Everything
DA-ANN	Deep Auto-encoder Artificial Neural Network
DBN	Deep Belief Networks
DICE	Deep Inference for Covariance Estimation
DR	Dead Reckoning
DSRC	Dedicated Short-Range Communication
EDCA	Enhanced Distribution Channel Access
eMBB	enhanced Mobile BroadBand
FCTA	Forward Cross Traffic Alert
FCWS	Forward Collision Warning System
FDMA	Frequency Division Multiple Access
FHSS	Frequency Hopping Spread Spectrum
FoV	Field of View
GFSK	Gaussian Frequency Shift Keying
GNSS	Global Navigation Satellite System
GPS	Global Positioning System
HetVNet	Heterogenous Vehicular Network
ID	IDentification
IMU	Inertial Measurement Unit
IoT	Internet of Things
IoV	Internet of Vehicles
IR	InfraRed
ITS	Intelligent Transport Systems
IVC	Inter-Vehicle Communication
LBAC	Laser Based Adaptive Cruise
LCA	Lane Change Assistance
LDPC	Low Density Parity Check
LDWS	Lane Departure Warning System
LiDAR	Light Detection and Ranging
LoS	Line of Sight
LRWPAN	Low Rate Wireless Personal Area Network
LSTM	Long Short-Term Memory
LTE	Long-Term Evolution
LTE-A	Long-Term Evolution-Advanced
MAC	Media Access Control
MEC	Multi-access Edge Computing
MEMS	Micro-ElectroMechanical System
MIVC	Multi-hop Inter-Vehicle Communication
ML	Machine Learning
mMTC	massive Machine Type Communication
mmwave	Millimetre Wave
NFC	Near Field Communication
NHSTA	National Highway Traffic Safety Administration
NLoS	Non-Line of Sight
NOMA	Non-Orthogonal Multiple Access
NS2	Network Simulator 2
OBU	On Board Unit
OICA	International Organisation of Motor Vehicle Manufacturer
OCB	Outside the Context of a Basic service set
OFDM	Orthogonal Frequency Division Multiplexing
O-QPSK	Orthogonal Quadrature Phase Shift Keying
OMNeT	Objective Modular Network Testbed in C++
PCW	Pedestrian Collision Warning
PDA	Personal Digital Assistance
PSFCH	Physical Sidelink Feedback CHannel
QAM	Quadrature Amplitude Modulation
QoS	Quality of Service
RADAR	Radio-Detection and Ranging
RCTA	Rear Cross Traffic Alert
RGB	Red, Green and Blue
RGBD	Red, Green, Blue, and Depth
RMSE	Root Mean Square Error
RNN	Recurrent Neural Networks
RSSI	Received Signal Strength Indicator
RSU	Road Side Unit
RTK	Real Time Kinetics
RVC	Roadside to Vehicle Communication
SAE	Society of Automotive Engineers
SC-FDMA	Single Carrier-Frequency Division Multiple Access
SIVC	Single-hop Inter-Vehicle Communication
SPA	Self-Parking Assistance
SRVC	Sparse Roadside to Vehicle Communication
SUMO	Simulation of Urban MObility
SVM	Support Vector Machine
TA	Trusted Authority
TDMA	Time Division Multiple Access
ToF	Time of Flight
TSR	Traffic Sign Recognition
UOR	Unknown Obstacles Recognition
uRLLC	ultra-Reliable Low Latency Communication
URVC	Ubiquitous Roadside to Vehicle Communication
UWB	Ultra-Wide Band
V2D	Vehicle to Device
V2G	Vehicle to Grid
V2I	Vehicle to Infrastructure
V2P	Vehicle to Pedestrian
V2R	Vehicle to Roadside
V2V	Vehicle to Vehicle
V2X	Vehicle to Everything
VANET	Vehicular Ad-hoc network
VPL	Vehicle Positioning and Localisation
WAVE	Wireless Access in Vehicular Environment
Wi-Fi	Wireless Fidelity
WLAN	Wireless Local Area Network
